# Sulfated Exopolysaccharides from *Porphyridium purpureum*: A Review of Extraction, Structural Characterization, and Chemical Properties for Advanced Applications

**DOI:** 10.12688/f1000research.173006.2

**Published:** 2026-01-23

**Authors:** Devi Maulina, Abdul Mun’im, Asep Bayu, Heri Setiawan, Najihah Mohd Hashim

**Affiliations:** 1 Faculty of Pharmacy, Universitas Indonesia, Cluster of Health Sciences Building, Depok, West Java, 16424, Indonesia; 2 National Metabolomics Collaborative Research Center, Faculty of Pharmacy, Universitas Indonesia, Depok, West Java, 16424, Indonesia; 3 Research Center for Vaccine and Drugs, Research Organization for Health, National Research and Innovation Agency (BRIN), Jl. Raya Jakarta-Bogor KM 46 Cibinong, Bogor, West Java, 16911, Indonesia; 4 Department of Pharmacy, Faculty of Medicine, University of Malaya, Kuala Lumpur, Federal Territory of Kuala Lumpur, 50603, Malaysia

**Keywords:** Porphyridium purpureum; sulfated exopolysaccharides; green extraction; membrane technology; structural characterization; bioactivity.

## Abstract

Microalgae are increasingly recognized as sustainable green cell factories capable of producing a various high-value bioactive compounds. Among them, the red microalga *Porphyridium purpureum* stands out for its unique ability to secrete sulfated exopolysaccharides (sEPS)—complex macromolecules enriched with sulfate and uronic acid groups that exhibit potent biological activities. These compounds function as natural antioxidants, immunomodulators, and antimicrobial agents, attracting substantial interest from the pharmaceutical, cosmetics, and functional food industries.

A targeted literature search was conducted across the Scopus, PubMed, Web of Science and ScienceDirect databases to identify studies focusing on the extraction, structural characterization, and applications of sulfated exopolysaccharides from *Porphyridium purpureum* between 2015 and 2025. Recent research has demonstrated rapid advancements in environmentally friendly extraction technologies, including membrane filtration, three-phase partitioning (TPP), ultrasound-assisted TPP (UATPP), and deep eutectic solvents (DES), which collectively enhance the yield, purity, and preserve the biological functionality of sEPS.

Furthermore, significant progress has been made in structural and mechanistic elucidation through advanced analytical and imaging techniques such as two-dimensional NMR spectroscopy, FTIR, methylation analysis, and transcriptomic profiling. These approaches have clarified how cultivation parameters and environmental stressors influence EPS biosynthesis, sulfation patterns, and biological activity.

Overall, this review provides an integrated and forward-looking perspective on the scientific advances and technological challenges surrounding *P. purpureum* sEPS, outlining future directions toward sustainable bioprocessing and industrial valorization of these high-value biomacromolecules.

## Introduction

*Porphyridium purpureum* is a unicellular red microalga recognized as a prolific source of sulfated exopolysaccharides (sEPS)—complex, high-molecular-weight biopolymers with distinctive physicochemical and biological properties. ^1, 2^ These compounds have attracted considerable attention for their multifunctional bioactivities **,** including antioxidant, antibacterial, antiviral, and immunostimulatory effects, which make them promising candidates for applications in biomedicine, food preservation, cosmetics, and aquaculture. ^3– 5^

The exceptional structural complexity of *P. purpureum* sEPS—defined by its diverse monosaccharide composition, extensive branching, and highly sulfated domains—serves as the molecular foundation for its broad functional versatility. ^6– 8^ Recent technological advances, such as membrane-based separation, solvent precipitation, ^9, 10^ and integrated green extraction methods such as *three-phase partitioning (TPP)*, *ultrasound-assisted TPP (UATPP),* ^11– 13^ and *deep eutectic solvents (DES)*, have significantly improved the efficiency, yield, and purity of sEPS recovery. ^14, 15^

Underlying these technological developments is the paradigm that extraction efficiency, structural integrity, and chemical modification are closely interdependent, collectively determining the functional properties and application potential of the resulting polymers. ^9, 16^ Theoretical insights from polymer chemistry and molecular biology have further explained how environmental factors and genetic regulation influence the biosynthesis and structural assembly of sEPS. ^17, 18^ For instance, advanced extraction approaches such as membrane filtration and UATPP are known to better preserve the molecular weight and sulfation—key determinants of bioactivity—while structural parameters such as the degree of sulfation and the branching pattern directly modulate the antioxidant, rheological, and immunostimulatory responses. ^19, 20^ Moreover, environmental conditions, including salinity and nitrogen availability, have been shown to regulate gene expression in the EPS biosynthetic pathway, thereby influencing yield, composition, and functional attributes. ^1, 17^

This review integrates these theoretical and empirical perspectives to provide a comprehensive synthesis of the extraction methodologies, structural characterization, chemical profiling, and emerging applications of *P. purpureum* sEPS. The review also identifies technological innovations, current limitations, and future research directions necessary to bridge the gap between laboratory-scale findings and industrial-scale use of this valuable microalgal biomaterial.

## Discussion

This review synthesizes studies published over the past 10 to 15 years, emphasizing recent advances in the extraction, structural characterization, and biofunctional evaluation of sEPS from *P. purpureum.* A targeted literature search was conducted across Scopus, PubMed, Web of Science, and ScienceDirect databases using relevant keywords to identify research on the production, extraction, structure elucidation, and applications of *P. purpureum* sEPS. The reviewed literature spans biotechnology, chemical engineering, and molecular biology, reflecting the interdisciplinary nature of sEPS research. This methodological foundation provides the context for the following discussion, which integrates experimental evidence and theoretical perspectives to analyze the efficiency of the extraction methods, the structure–function relationships, and the environmental modulation of the sEPS bioactivity.

### Extraction methods: Efficiency and innovation

The extraction of sEPS from *P. purpureum* has progressed substantially over the past decade. ^9, 20^ Early studies relied on simple solvent precipitation using ethanol, methanol, or isopropanol to recover crude polysaccharides from the culture medium. This classical method remains widely used due to its simplicity and low capital demand, yet it frequently causes partial desulfation, co-precipitation of proteins, and inconsistency in the molecular weight, which ultimately reduce the functional quality of the recovered polymers. ^19^

Advances in downstream engineering introduced membrane-based separation systems such as ultrafiltration and diafiltration. These approaches enable better control of the molecular size and purity, generating sEPS fractions with high sulfate content and stable rheological behavior. The main challenge arises from membrane fouling, which limits the flux and operational efficiency during continuous or large-scale production. ^16^

Recent innovations emphasize environmentally responsible and integrated extraction strategies. Three-phase partitioning (TPP), ultrasound-assisted TPP (UATPP), and deep eutectic solvent (DES)-based methods have demonstrated remarkable ability to recover and purify sEPS simultaneously while minimizing solvent consumption and energy demand. ^21^ TPP and UATPP consistently deliver high yield and excellent bioactivity retention, whereas DES provides a greener alternative that maintains the molecular weight and sulfation degree. These methods represent a clear shift from conventional solvent-based extraction toward high-performance, sustainable bioprocesses suitable for industrial adaptation. ^20, 21^

**Figure 1.**
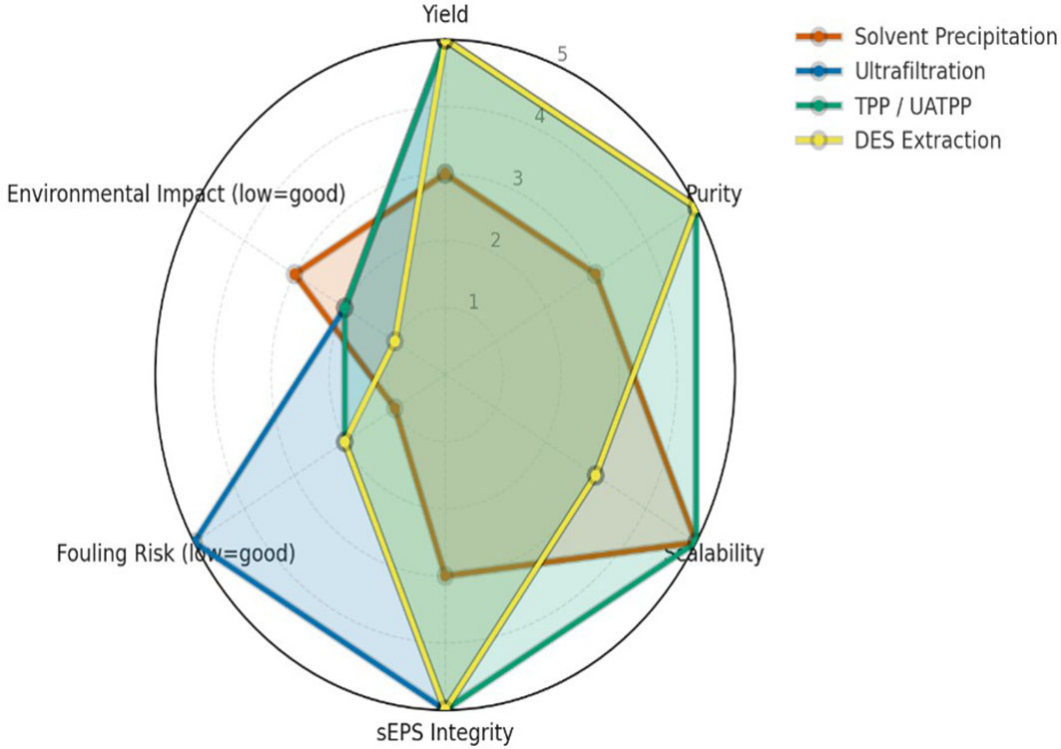
Performance comparison of sEPS extraction methods across key parameters. Radar plot comparing the performance of four extraction strategies for sEPS from *P. purpureum* across six criteria (yield, purity, scalability, structural integrity, fouling risk, environmental impact), scored on a harmonized 1–5 scale. Higher values indicate superior performance, with fouling and environmental scores inversely weighted. TPP/UATPP and DES show stronger preservation of polymer structure and sustainability, while ultrafiltration excels in purity and solvent precipitation remains simplest but less consistent.

**Figure 2.**
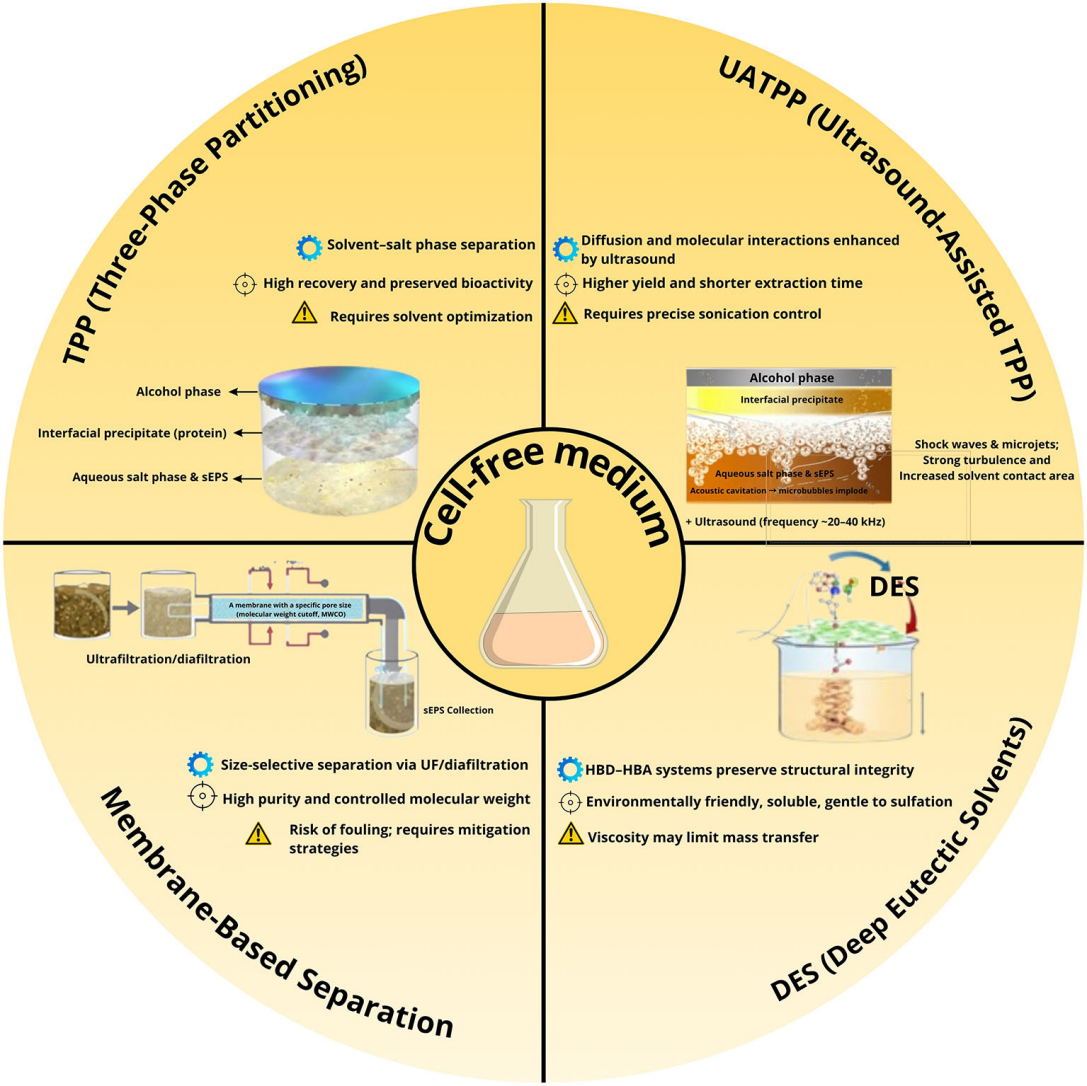
Comparative overview of green and integrated extraction strategies for sEPS from *P. purpureum.* Summarizing mechanistic principles (⚙), key performance outcomes (⊕), and practical considerations (⚠) associated with Three-Phase Partitioning (TPP), Ultrasound-Assisted TPP (UATPP), Deep Eutectic Solvents (DES), and membrane-based separation techniques.

Comparative evaluation across various extraction techniques highlights significant differences in yield, purity, scalability, fouling tendency, and environmental impact. The semi-quantitative assessment ( Figure 1) summarizes these parameters, while a complementary infographic ( Figure 2) outlines the mechanistic principles and operational considerations behind the leading green extraction technologies. Among them, TPP/UATPP and DES extraction consistently outperform solvent precipitation and single-step ultrafiltration in terms of both product quality and eco-efficiency.

Industrial implementation of intensified green extraction still faces technical and economic barriers. TPP/UATPP demands precise control of solvent phase ratios, ultrasound energy, and solvent recycling to achieve continuous sustainable production. DES-based systems encounter viscosity-limited mass transfer and relatively high raw-material cost, and their techno-economic and life-cycle performance remains under-documented. Progress in these areas requires integrated research that combines cost modeling, solvent regeneration, and predictive fouling-resilient downstream design to strengthen the feasibility of future microalgal biorefineries.

**Figure 3.**
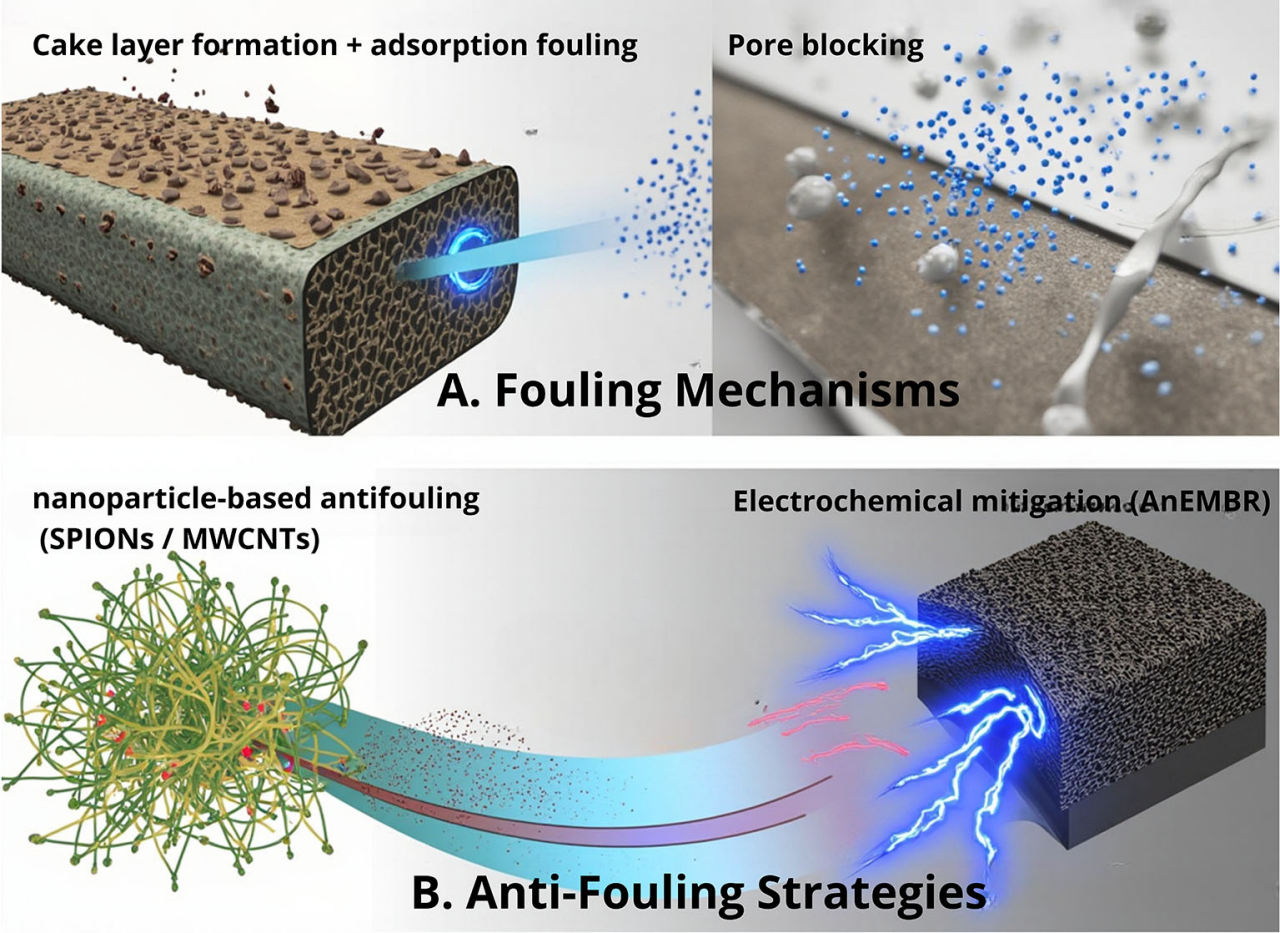
Membrane fouling mechanisms and anti-fouling strategies during sEPS separation. (A) Fouling mechanisms during microalgal sEPS filtration include cake layer formation, adsorption fouling, and pore blocking. (B) Anti-fouling strategies target sEPS–membrane interactions through nanomaterial-based surface modification and electrochemical approaches to reduce deposition and improve filtration efficiency.

Membrane-based separation plays a vital role in the downstream recovery of sEPS from microalgae; however, fouling driven by the highly charged and viscous characteristics of sEPS—including adsorption fouling, pore blocking, and cake layer formation—remains a major bottleneck that causes a severe flux decline and increases the operational cost. As illustrated in Figure 3, several engineering strategies have been explored to mitigate these surface and pore-scale interactions. Calcium ion addition modulates the sEPS surface charge and aggregation behavior, helping to minimize pore blockage and adsorption when applied at optimal concentrations. ^22^ Nanomaterial-based surface modification (e.g., SPIONs and MWCNTs) enhances the membrane hydrophilicity and reduces foulant adhesion, thereby improving the molecular transport and filtration stability, although at a higher fabrication cost. ^23, 24^ Meanwhile, electrochemical membrane bioreactors (AnEMBR) apply electric fields to suppress the deposition of negatively charged macromolecules and slow cake formation, making this approach particularly attractive for anaerobic or hybrid separation systems. ^25^ Collectively, these strategies demonstrate promising solutions to improve the membrane efficiency and support scalable sEPS purification.

### Structural characterization: complexity and bioactivity

Advanced structural studies have provided detailed insights into the chemical architecture of sEPS secreted by *Porphyridium purpureum.* Spectroscopic evidence, primarily from 1D and 2D nuclear magnetic resonance (NMR) and methylation analyses, reveals that these polymers possess a nearly linear backbone composed of (1→2)- or (1→4)-linked D-xylopyranosyl, (1→3)-linked L-galactopyranosyl, and (1→3)-linked D-glucopyranosyluronic acid residues. ^26– 28^

Sulfate groups are typically attached at specific positions—O-6 of glucose and O-3/O-4 of xylose—while branching occurs at xylose and galactose residues, forming a flexible yet stable molecular framework. ^26, 29^ This highly ordered but variably branched structure contributes to the polymer’s anionic charge density, hydration capacity, and gel-forming ability, which are critical for its rheological and biological performance.

Comparative NMR analyses across the *Porphyridium* strains indicate that the distribution of sulfate esters and uronic acid units governs not only solubility and viscosity but also the metal-binding capacity—a property relevant to biomedical and environmental applications. Moreover, the predominance of acidic monosaccharides and sulfation at the terminal residues enhances the formation of extended hydrated networks, supporting stability under extreme salinity, temperature, and pH conditions. ^26, 28^ Together, these molecular configurations establish *P. purpureum* sEPS as a structurally complex biopolymer with tunable physicochemical properties ideal for functional and therapeutic use.

**Figure 4.**
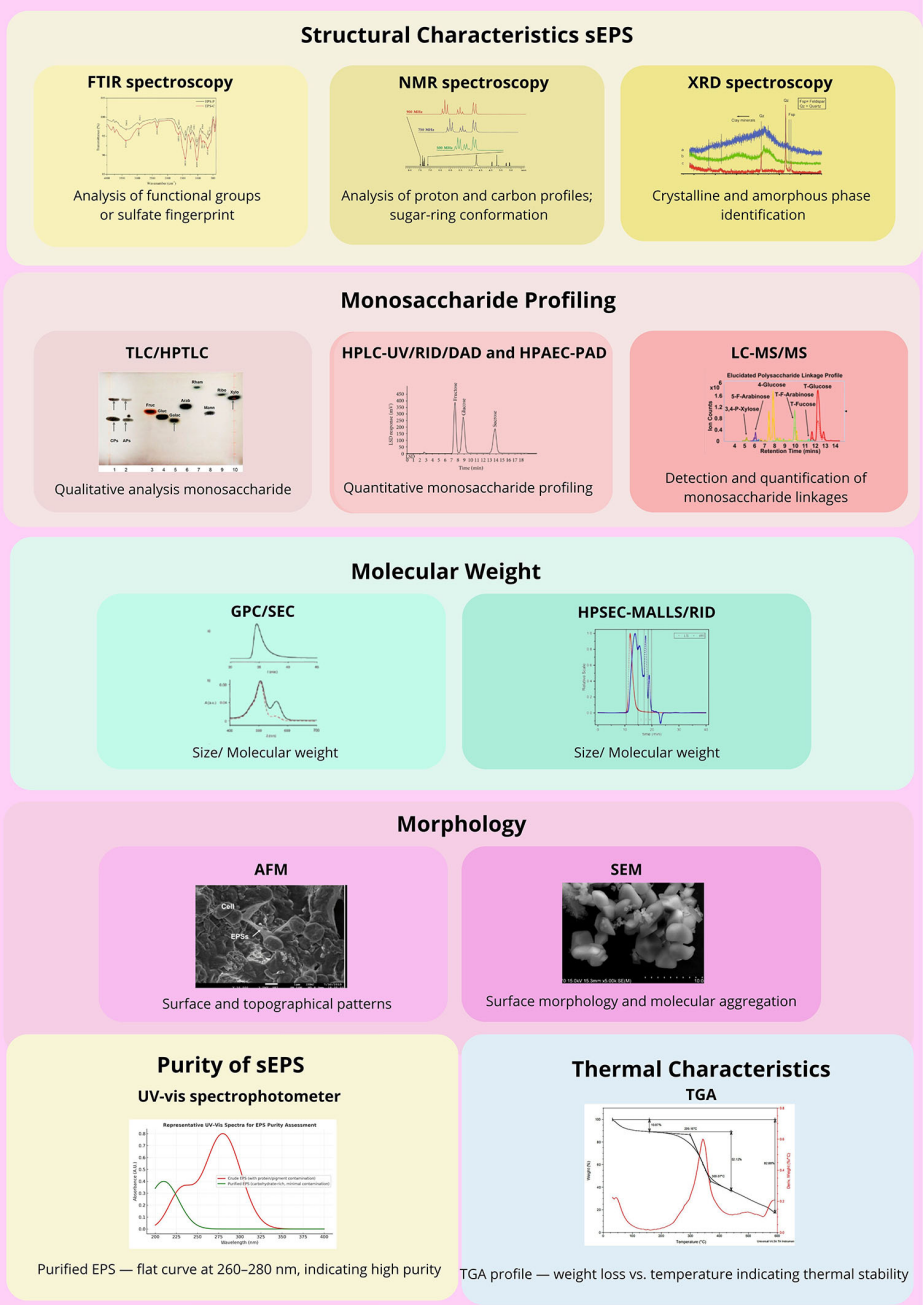
Analytical characterization techniques for sEPS from *P.purpureum.* Abbreviations: FTIR – Fourier Transform Infrared; NMR – Nuclear Magnetic Resonance; XRD – X-ray Diffraction; TLC – Thin Layer Chromatography; HPTLC – High-Performance Thin Layer Chromatography; HPLC – High-Performance Liquid Chromatography; RID – Refractive Index Detector; DAD – Diode Array Detector; HPAEC-PAD – High-Performance Anion Exchange Chromatography with Pulsed Amperometric Detection; LC–MS/MS – Liquid Chromatography–Tandem Mass Spectrometry; GPC/SEC – Gel Permeation/Size Exclusion Chromatography; HPSEC–MALLS/RID – High-Performance Size Exclusion Chromatography with Multi-Angle Laser Light Scattering/Refractive Index Detector; AFM – Atomic Force Microscopy; SEM – Scanning Electron Microscopy; UV–Vis – Ultraviolet–Visible Spectrophotometer; TGA – Thermogravimetric Analysis.

To further substantiate these structural insights, a combination of analytical and spectroscopic techniques was applied to characterize the sEPS comprehensively ( Figure 4). ^30– 32^ Methylation analysis and partial hydrolysis remain pivotal for determining the linkage types and glycosidic configurations, thereby revealing the branching patterns and backbone heterogeneity. When coupled with Smith degradation, these classical degradation-based methods enable detailed mapping of the monosaccharide residues and substitution sites within the macromolecule. ^33^

NMR spectroscopy ( ^1^H, ^13^C, and 2D-NMR) provides comprehensive information on the structural motifs, substitution degrees, and sugar-ring conformations, while FTIR and XRD analyses identify the functional groups and crystalline–amorphous transitions. Comparative omics-assisted studies have linked sulfate-ester distribution and uronic acid content with the regulation of biosynthetic genes under salinity or nutrient stress. ^30^ Such multi-level analyses highlight the adaptive molecular strategies that generate highly sulfated and thermally stable polysaccharides in red microalgae.

From a biochemical perspective, monosaccharide profiling using chromatographic and spectrophotometric techniques (HPLC-UV/RID/DAD, HPAEC-PAD, LC–MS/MS, and HPSEC) consistently identified xylose, galactose, glucose, and glucuronic acid as the dominant constituents. ^7^ The relative abundance of these sugars defines the polymer’s identity and functional properties. Uronic acids and sulfate esters impart a high negative-charge density, conferring excellent hydration and gel-forming ability—key factors in antioxidant and immunomodulatory functions. ^31^

Molecular-weight analysis via GPC/SEC typically shows ranges of 8 × 10 ^5^–3 × 10 ^6^ Da, correlating with desirable viscosity, film-forming potential, and stability. ^34, 35^ Complementary gravimetric and titrimetric assays quantify sulfate and uronic acid content, parameters directly associated with antioxidant potency and biological activity. Functional assessments confirm that DPPH and hydroxyl-radical scavenging activities can reach up to 90% inhibition, while TGA and DSC analyses reveal excellent thermal stability under industrial processing conditions. ^36^ Microscopic examinations such as AFM and SEM further visualized the surface morphology and topographical organization of the purified EPS, validating its uniformity and aggregation behavior.

Collectively, these results emphasize that integrating chemical, spectroscopic, and bioactivity data provides a robust structure–function framework for *P. purpureum* sEPS. The degree of sulfation, molecular-weight distribution, and monosaccharide composition jointly determine its physicochemical versatility and therapeutic potential, reinforcing its promise as a bioactive polysaccharide suitable for pharmaceutical, food, and cosmetic applications.

### Chemical properties: Structure–function relationships

The chemical and biological functions of *P. purpureum* sEPS are closely linked to their structural organization and compositional diversity. These polymers generally exhibit high molecular weight (6.6–8.3 × 10 ^6^ Da), substantial sulfate content (8–20%), and notable uronic acid fractions (10–15%), with balanced ratios of xylose, galactose, and glucose. ^34, 35^ This composition provides both mechanical resilience and biochemical reactivity, supporting their multifunctional behavior. The degree of sulfation serves as a key determinant of bioactivity—higher sulfation enhances the antioxidant potential, DPPH radical scavenging activity, and thermal stability, whereas insufficient sulfation reduces the reactivity toward reactive oxygen species. ^37^

From a biochemical standpoint, anionic sulfate and carboxyl groups mediate electrostatic interactions with proteins, metal ions, and biological membranes, forming the mechanistic basis for the anticoagulant, antiviral, and immunostimulatory effects widely observed across *Porphyridium* species. Collectively, these observations establish a direct structure–function paradigm, emphasizing that both chemical complexity and environmental modulation are essential to optimize sEPS bioactivity for pharmaceutical, nutraceutical, and cosmetic applications. ^1^

Building upon these chemical and structural insights, the following section examines how environmental and metabolic cues orchestrate the biosynthetic regulation of these sulfated exopolysaccharides, highlighting the dynamic interplay between physiological adaptation and molecular control in *P. purpureum.*

### Environmental and metabolic regulation

Environmental and metabolic factors significantly influence the biosynthesis of sEPS in *P. purpureum.* Changes in salinity, nutrient availability, and light quality can shift carbon allocation, affect cellular redox balance, and regulate the expression of genes involved in sulfate activation and glycosylation pathways. When algae face nitrogen deficiency or osmotic stress, the carbon flow initially directed toward protein synthesis is diverted toward carbohydrate formation. This metabolic shift drives the increased production of sulfate-rich extracellular polysaccharides. The activity of key enzymes such as ATP sulfurylase and APS kinase also increased significantly. These two enzymes are responsible for producing 3’-phosphoadenosine-5’-phosphosulfate (PAPS), a universal sulfate donor that is key in the sulfotransferase reaction that forms the complex and bioactive sEPS structure. ^38, 39^

Genome sequencing of *P. purpureum* revealed a compact but function-rich genome (19.7 Mbp, 8,355 predicted genes) containing a diverse repertoire of carbohydrate-active enzymes (CAZymes), including glycosyltransferases, sulfotransferases, and carbohydrate-sulfate esterase families implicated in the modification of the cell-wall and extracellular polysaccharides. Phylogenetic analysis indicates that several of these genes originated from horizontal gene transfer (HGT) events from prokaryotic and chromalveolate lineages, suggesting the adaptive enrichment of sulfur metabolism and extracellular matrix biosynthesis in marine environments. ^40^ In addition, the absence of canonical formylglycine-dependent sulfatases (FGly-SULFs) and sulfatase-modifying factors (SUMFs) in red algae implies that *P. purpureum* relies primarily on sulfotransferase-driven sulfation rather than enzymatic desulfation, resulting in stable, highly sulfated polysaccharide structures. ^41^

The environmental modulation of sulfation pathways is strongly dependent on light and salinity. Optimization studies have demonstrated that white light and moderate NaCl or KH _2_PO _4_ enrichment maximize both biomass and sEPS productivity, whereas calcium ions (CaCl _2_) enhance polymer crosslinking and extracellular secretion efficiency. ^42^ These responses are consistent with the role of reactive oxygen species and ionic stress as signaling cues that upregulate the transcription of stress-responsive genes, including those encoding nucleotide-sugar epimerases and glycosyltransferases involved in the branching and sulfation of EPS chains. The resulting increase in the degree of polymer sulfation improves the bioactivity and confers enhanced antioxidative and rheological properties.

At the molecular level, comparative “red and green” sulfur metabolism models indicate that sulfate assimilation in *P. purpureum* follows the canonical two-step activation through ATP sulfurylase and APS kinase, as established in other photosynthetic eukaryotes. ^39^ The synthesis of PAPS links environmental sulfur availability to cellular regulation: oxidative stress and nutrient deprivation activate ATP sulfurylase, while the reduction of APS kinase under high redox potential diverts flux from primary assimilation (cysteine synthesis) toward secondary sulfur metabolism and extracellular sulfation. The coupling of these regulatory nodes enables *P. purpureum* to dynamically balance growth and defense, modulating the sEPS composition in response to environmental perturbations.

However, direct gene-level and omics-integrated data for *P. purpureum* remain scarce. While transcriptomic profiles from related red algae show coordinated upregulation of sulfotransferases, glycosyltransferases, and nucleotide-sugar epimerases under stress, the absence of comprehensive proteomic and metabolomic mapping limits the mechanistic understanding. Future studies integrating transcriptomics, proteomics, and metabolomics under controlled environmental stimuli will be essential to decipher the regulatory networks governing sEPS biosynthesis. Such insights would enable the targeted metabolic engineering of *P. purpureum* for enhanced sEPS yield and tailored structural functionality.

### Chemical properties and functional activities

Building upon the established structure–function and regulatory insights, the measurable chemical characteristics of *P. purpureum* sEPS reveal critical connections to their physicochemical stability and biological performance. High molecular weight and dense sulfation contribute to the exceptional rheological properties, radical-scavenging activity, and heat resistance, while uronic acids enhance the metal ion chelation and oxidative protection. Balanced monosaccharide ratios further improve the solubility and gel strength—key attributes for industrial processing and formulation.

Environmental factors, notably nitrogen availability and salinity, dynamically shape these characteristics by modulating the biosynthetic enzyme activity and sulfation patterns.

This interplay between structure, chemistry, and metabolism underpins the antioxidant, immunomodulatory, and stability-enhancing properties that make *P. purpureum* sEPS an attractive biopolymer for multi-sectoral applications. The measurable chemical characteristics of the sEPS produced by *P. purpureum* show a strong correlation with their physicochemical stability and biological performance. Variations in environmental factors, particularly salinity and nitrogen concentration, trigger distinct structural and functional adaptations within the polymer network. The ionic composition of the culture medium plays a crucial role in modifying the sulfation patterns and monosaccharide profile of the sEPS. Increasing the salinity up to 50 g L ^−1^ NaCl promotes selective sulfation on xylose and galactose residues, resulting in stronger ionic crosslinking and higher structural rigidity. ^30^ These modifications enhance the polymer’s resistance to thermal degradation and improve its stability under ionic conditions involving both monovalent and divalent cations. Consequently, *P. purpureum* polysaccharides possess high potential for application in cosmetic and pharmaceutical formulations that require strong physicochemical resilience. ^7^

Nitrogen availability determines the biochemical balance among the carbohydrate, protein, and uronic acid fractions within the EPS matrix. Nitrogen-enriched cultures yield higher amounts of EPS and exhibit an increased carbohydrate-to-protein ratio. In contrast, nitrogen limitation triggers stress-induced carbon redistribution, altering the branching levels and uronic acid content. These compositional shifts fine-tune the viscosity, charge density, and solubility of sEPS, thereby influencing their stability and processability. ^17, 34^

The antioxidant potential of *P. purpureum* sEPS primarily arises from their high sulfate substitution and uronic acid content. These anionic groups act as effective electron donors, neutralizing reactive oxygen species such as hydroxyl and DPPH radicals. Cultures grown under higher salinity demonstrated stronger radical-scavenging capacities, indicating that environmental stress enhances the number of redox-active groups within the polymer backbone. This property supports the use of sEPS as a natural antioxidant agents in dermatological formulations and nutraceutical products. ^1, 35^

Immunostimulatory effects have also been observed through the activation of B lymphocytes and macrophages. The structural complexity, characterized by a high degree of sulfation and densely branched glycosidic linkages, facilitates electrostatic interactions with immune receptors, thereby enhancing cytokine release and humoral responses. These findings highlight the potential of *Porphyridium*-derived sEPS as functional biomolecules for supporting immune function in both human health and aquaculture applications. ^30^

The protective role of sEPS against environmental stress represents another important characteristic. Under salt stress or nutrient limitation, *P. purpureum* activates polysaccharide biosynthetic pathways that lead to the accumulation of a viscous extracellular matrix. This matrix maintains osmotic balance and protects cells from oxidative damage, reinforcing the dual structural and ecological role of sEPS as both a protective barrier and an adaptive mechanism. ^18^

The sEPS matrix of *P. purpureum* was dominated by xylose, galactose, and glucose, accompanied by uronic acids and a high degree of sulfation (8–20%). Environmental factors influence the sulfation profile and the overall polymer architecture. Elevated salinity enhances sulfate incorporation at specific hydroxyl positions, whereas nitrogen variation modulates the branching density and structural organization. This chemical plasticity directly contributes to improved thermal stability, ionic tolerance, and bioactivity, forming the mechanistic basis for their multifunctional potential. A comprehensive understanding of the structure–function relationships of sEPS enables the rational optimization of culture conditions to produce high-value compounds suitable for pharmaceutical, nutraceutical, and cosmeceutical applications. ^1, 30, 34, 35^

The synergistic combination of these chemical parameters defines the adaptive molecular design of *P. purpureum* sEPS, reflecting its ecological resilience and biotechnological potential.

These physicochemical and functional attributes form the foundation for diverse biomedical and industrial applications, as outlined in the following section.

The summarized chemical characteristics and corresponding biofunctional implications of the *P. purpureum* EPS are presented in Table 1, highlighting the relationships between the molecular weight, sulfate and uronic acid content, and their antioxidant and immunomodulatory activities under different environmental conditions.

**Table 1.** Chemical properties and functional activities of sEPS from *P. purpureum.*

Property	Typical value	Functional implication	Modulated by	Ref
Molecular Weight	6.6–8.3 × 10 ^6^ Da	Determines rheological behavior and bioactivity; higher molecular weight enhances viscosity and film-forming properties	Nitrogen availability, extraction method	^34, 35^
Sulfate Content	~21.6% (variable)	Contributes to antioxidant and immunostimulatory activity through increased negative charge density	Salinity, degree of sulfation	^1, 30^
Uronic Acid Content	Significant (10–15%)	Improves antioxidant capacity and polymer stability via metal ion chelation	Nitrogen concentration, salinity	^1, 30^
Thermal Stability	High (stable up to 200°C)	Ensures suitability for food and industrial processing applications	Degree of sulfation and cross-linking	^42^
Antioxidant Activity	DPPH radical scavenging up to 92%	Indicates strong potential for food preservation and biomedical formulations	Sulfation level, molecular weight	^37, 43^

### Biomedical and industrial applications

***Antioxidant and antimicrobial bioactivity***

sEPS from *Porphyridium* species act as potent natural antioxidants and antimicrobials. Their sulfate groups and uronic acids contribute to radical scavenging via hydrogen donation and metal chelation, protecting biological substrates from oxidative degradation. ^35^ In food systems, these polymers delay lipid peroxidation and protein carbonylation, as shown in refrigerated meat, extending shelf life by suppressing microbial proliferation and oxidative rancidity. ^3^ Their antibacterial activity targets both Gram-positive and Gram-negative bacteria, likely through electrostatic interaction between negatively charged sulfate moieties and microbial membranes, impairing nutrient transport and cell integrity.

***Immunostimulant and aquaculture applications***

Immunomodulatory potential has been validated *in vivo* using zebrafish embryos and Pacific white shrimp ( *Litopenaeus vannamei*). *P. purpureum* EPS was proven nontoxic and enhanced shrimp hemocyte counts, phagocytic activity, and respiratory burst, providing protection against *Vibrio harveyi* infection. ^4^ These findings support its application as a sustainable immunostimulant or vaccine adjuvant alternative in aquaculture to reduce antibiotic dependence and disease outbreaks.

***Anticancer potential***

Anticancer activity was predominantly associated with low-molecular-weight sEPS fractions (approximately 300–550 kDa) from *Porphyridium marinum*, generated via high-pressure homogenization (HPH), which exhibited enhanced antiproliferative effects against murine breast cancer cells, with up to 55% reduction in cell viability. ^5^ The enhanced anticancer activity was correlated with lower viscosity and increased accessibility of reactive sulfate and uronic acid sites, suggesting that molecular tailoring can optimize pharmacological potency.

***Food preservation and functional additives***

The antioxidant and antibacterial actions of *Porphyridium* sEPS provide a natural biopreservative alternative for perishable foods. ^3^ When incorporated at 0.5%–2% (w/w) in minced meat, it markedly decreased lipid oxidation and microbial load during refrigerated storage. Its high water-holding and oil-binding capacities improve the texture and reduce syneresis, indicating its suitability as a stabilizer in emulsions and high-fat formulations.

***Rheological and thermal stability***

Rheological analysis revealed that *P. purpureum* EPS solutions (1%) maintain viscoelasticity and viscosity under wide temperature and salinity ranges, even in the presence of mono- and divalent cations such as Na ^+^ and Ca ^2+^. ^7^ His exceptional stability—linked to the ionic cross-linking among sulfate groups—surpasses many plant polysaccharides and makes sEPS valuable as thickeners, gelling agents, and stabilizers in the food, cosmetic, and pharmaceutical industries, especially under extreme pH or temperature processing.

Although a high degree of sulfation contributes to the rheological strength of sEPS, several studies indicate that reduced molecular weight and lower viscosity can enhance biological activity by improving the accessibility of sulfate and uronic acid groups. ^5^ These findings highlight that the biofunctional performance of sEPS depends on a balance between sulfation, molecular weight, and rheological behavior, depending on the intended industrial or biological application.

***Cosmetic formulations***

In the cosmetic sector, *Porphyridium* sEPS serve as multifunctional biopolymers providing hydration, film-forming, and anti-aging benefits. Their polyanionic structure forms a moisturizing matrix on the skin surface, retaining water while shielding against oxidative and microbial stress. Combined with their rheological stability and natural origin, these polymers are now considered promising substitutes for synthetic thickeners in high-value dermocosmetic formulations. ^7, 35^

***Green processing and molecular engineering***

Emerging green technologies such as three-phase partitioning (TPP), ultrasound-assisted extraction (UATPP), and HPH enable controlled depolymerization, adjusting the molar mass and bioactivity without the use of toxic solvents. ^5^ This allows the industry to tune the viscosity and biofunctionality for specific sectors—pharma (injectable immunostimulants), food (emulsifiers), and cosmetics (bio-film moisturizers)—while maintaining the environmental sustainability of microalgal production.

***Integrative perspective***

The broad biomedical and industrial spectrum of *P. purpureum* sEPS stems from its unique chemical architecture—a high-molecular-weight heteropolysaccharide enriched in sulfate, uronic acids, and galactose/xylose residues conferring redox activity, ionic cross-linking, and biocompatibility. These characteristics underpin a circular bioeconomy potential: sustainable microalgal cultivation, clean extraction, and multifunctional biopolymer use across the food preservation, health care, aquaculture, and cosmetics sectors.

In summary, *Porphyridium purpureum* sEPS function as bioactive antioxidants, immunostimulants, and rheologically robust biopolymers—bridging pharmaceutical efficacy with industrial applicability through their chemical versatility and ecological sustainability.

### Research gaps and future perspectives

Although significant progress has been made in the extraction and structural elucidation of *Porphyridium purpureum* sEPS, several critical challenges remain before their full-scale industrial application can be achieved. Current cultivation systems still produce relatively low EPS titers, limiting the economic feasibility for industrial-scale production. Efficient coupling the between cultivation, extraction, and purification stages also remains difficult due to membrane fouling, co-extraction of impurities, and product losses during downstream processing. Moreover, the lack of comprehensive multi-omics data hampers the targeted enhancement of EPS biosynthesis and limits an in-depth understanding of gene-level regulation controlling sulfation and polymer assembly. Variations in analytical protocols—particularly in molecular weight determination, sulfate quantification, and rheological profiling—further complicate cross-study comparison and reduce data reproducibility.

To address these limitations, future research should prioritize integrated, multi-stage cultivation–extraction systems that use real-time process monitoring and AI-based control to dynamically regulate environmental and metabolic parameters. In parallel, metabolic engineering and adaptive cultivation strategies should be developed to enhance the carbon flux toward EPS biosynthesis and to control the structural diversity. Combining these advances with high-resolution analytical and omics-based characterization will enable the precise tailoring of sEPS composition and functionality for pharmaceutical, cosmetic, and food applications.

Bridging these research gaps will be crucial to transform *P. purpureum* sEPS from a promising experimental innovation into a sustainable, large-scale bioresource aligned with circular bioeconomy principles.

## Conclusion

sEPS derived from *Porphyridium purpureum* represent a promising class of multifunctional biopolymers with broad applicability in biomedicine, food, cosmetics, and aquaculture. Recent advances in extraction technologies—particularly membrane-based and green approaches—have markedly enhanced the product yield and purity. In parallel, sophisticated analytical techniques have deepened the structural understanding, revealing the intricate sulfation patterns, branching configurations, and high molecular weights that govern the physicochemical stability and biological activity of these polymers.

Environmental modulation and genetic regulation have also emerged as key factors influencing sEPS biosynthesis, offering new avenues to optimize productivity and tailor specific bioactivities. Nevertheless, major challenges persist in achieving large-scale production, integrating cultivation–extraction processes, and fully deciphering the structure–function relationships that determine performance across applications.

Addressing these limitations will require a synergistic framework combining integrated bioprocessing, advanced molecular analyses, and innovative bioengineering strategies. Such integration will be pivotal for translating laboratory-scale advances into sustainable industrial and clinical applications. The review demonstrates that *P. purpureum* EPS are obtained through innovative extraction methods and possess complex structures directly linked to their bioactive properties. Although their application potential is substantial, overcoming scale-up and process challenges through integrated research and technological innovation remains essential for successful industrial translation.

## Data Availability

No data are associated with this article.
