# Supplementary material for: Sulfated Exopolysaccharides from Porphyridium purpureum: A Review of Extraction, Structural Characterization, and Chemical Properties for Advanced Applications
Source: F1000Res. 2026 Jan 23;14:1323. Originally published 2025 Nov 27. [Version 2] doi: 10.12688/f1000research.173006.2 (PMC12988356; doi:10.12688/f1000research.173006.2)
Supplement: Peer review history

# Reviewer report: Reviewer response for version 2

**DOI:** 10.5256/f1000research.195162.r459656

**Published:** 2026-03-13

**Authors:** Wassim Guermazi

**Affiliations:** 1 University of Sfax Tunisia, Sfax, Tunisia

I have read the entire manuscript, and it is consistent and very interesting for the field of biotechnology, particularly regarding the extraction of EPS, which are of great interest in several fields. The authors provide in-depth information and compare the effects of different extraction methods on both the yield and the quality of EPS.

 I have also read the comments of the first reviewer. The manuscript has improved compared to the first version; however, the authors have not taken into account all the points raised by the reviewer, Mayra Alejandra Lopez.

 However, I have some minor comments:

- The manuscript presents good and informative figures. However, it is not clear whether these figures were created by the authors. If not, the authors should add the appropriate references for all figures. For the tables, the information is well referenced.
- The citation of Figure 3 is indicated in the first section of the Discussion (“Extraction methods: Efficiency and innovation”), but the figure appears later in the following section. Therefore, this figure should be included in the first section, possibly after Figure 2. In addition, it would be better to place Table 1 at the end of the corresponding section.
- There are still some issues with abbreviations, such as TGA and DSC. TGA should be defined at its first occurrence in the text. On page 7, what does DSC mean? Please define it when it first appears.
- Page 10: please add references supporting the following statement: “Cultures grown under higher salinity demonstrated stronger radical-scavenging capacities, indicating that environmental stress enhances the number of redox-active groups within the polymer backbone.” The authors could, for example, cite the following reference: “Mansour, F. B., Guermazi Wassim, Chamkha, M., Bellassoued, K., Ben Salah, H., Harrath, A. H., Aldahmash, W., Rahman, M. A., & Ayadi, H. (2024). Bioactive Potential of the Sulfated Exopolysaccharides From the Brown Microalga Halamphora sp.: Antioxidant, Antimicrobial, and Antiapoptotic Profiles. Analytical Science Advances, 5(9–10), e202400030”. This study investigates the antioxidant potential of EPS extracted from the microalga Halamphora sp. cultivated under high salinity (80 PSU). (Ref 1)

Is the review written in accessible language?

Yes

Are all factual statements correct and adequately supported by citations?

Yes

Are the conclusions drawn appropriate in the context of the current research literature?

Yes

Is the topic of the review discussed comprehensively in the context of the current literature?

Yes

Reviewer Expertise:

biomolecules from microalgae

I confirm that I have read this submission and believe that I have an appropriate level of expertise to confirm that it is of an acceptable scientific standard.

## References

[1] : Bioactive Potential of the Sulfated Exopolysaccharides From the Brown MicroalgaHalamphora sp.: Antioxidant, Antimicrobial, and Antiapoptotic Profiles. *Analytical Science Advances* .2024;5(9-10) : 10.1002/ansa.202400030 10.1002/ansa.202400030 PMC1151954439479574

# Reviewer report: Reviewer response for version 2

**DOI:** 10.5256/f1000research.195162.r452800

**Published:** 2026-01-30

**Authors:** Mayra Alejandra López-Ortega

**Affiliations:** 1 Universidad Autonoma del Estado de Hidalgo, Pachuca, Hidalgo, Mexico

The observations have been considered and appropriately addressed. I have no further comments.

Is the review written in accessible language?

Yes

Are all factual statements correct and adequately supported by citations?

Yes

Are the conclusions drawn appropriate in the context of the current research literature?

Yes

Is the topic of the review discussed comprehensively in the context of the current literature?

Yes

Reviewer Expertise:

Microbial polysaccharides

I confirm that I have read this submission and believe that I have an appropriate level of expertise to confirm that it is of an acceptable scientific standard.

# Reviewer report: Reviewer response for version 1

**DOI:** 10.5256/f1000research.190780.r442751

**Published:** 2026-01-08

**Authors:** Mayra Alejandra López-Ortega

**Affiliations:** 1 Universidad Autonoma del Estado de Hidalgo, Pachuca, Hidalgo, Mexico

I have reviewed your intriguing and very interesting review article on the current state of the art of sulfated extracellular polysaccharides produced by the microalga *Porphyridium purpureum*. Overall, it is a well-described and well-argued article. I have some suggestions that could be considered to improve the manuscript.

 Page 6. Paragraph 5. Sulfated exopolysaccharides (sEPS) are redefined; if they have already been defined, it is not necessary to do so again. Simply writing sEPS is sufficient.

 Page 8. As in the previous comment about the acronym sEPS, it is not necessary to define it again.

 Page 10. “Their antibacterial activity targets both gram-positive and gram-negative bacteria…” Gram must be written with a capital letter at the beginning, as it comes from a proper noun.

 Page 10. Anticancer potential… it would be interesting to mention the low molecular weights of the sEPS fractions that have this biological activity. The idea and the point are interesting, but it remains ambiguous if it is not mentioned.

 Throughout the document, the authors discuss how the presence of sulphated groups enhances the rheological properties of sEPS and that this also promotes interesting biological activities. However, some literature, such as *'Potential of exopolysaccharide from Porphyridium marinum to contend with bacterial proliferation, biofilm formation, and breast cancer,'* reveals that low viscosity improves the accessibility of sulphated and uronic groups, thereby enhancing their pharmacological potential. Therefore, it would be interesting to include a paragraph about these rheological qualities, perhaps viscosity values, and their correlation with bioactivities.

Is the review written in accessible language?

Yes

Are all factual statements correct and adequately supported by citations?

Yes

Are the conclusions drawn appropriate in the context of the current research literature?

Yes

Is the topic of the review discussed comprehensively in the context of the current literature?

Yes

Reviewer Expertise:

Microbial polysaccharides

I confirm that I have read this submission and believe that I have an appropriate level of expertise to confirm that it is of an acceptable scientific standard, however I have significant reservations, as outlined above.

# Response

**Published:** 2026-01-11

**Authors:** Devi Maulina

**Affiliations:** Faculty of Pharmacy, Universitas Indonesia, Depok, West Java, Indonesia

We sincerely thank the Reviewer for the thorough evaluation and the constructive comments, which have helped us to improve the clarity, consistency, and scientific depth of the manuscript. All suggestions have been carefully addressed as detailed below.

**Response to Comments 1 and 2**: Consistent Use of the sEPS Acronym

 Redundant definitions of sulfated exopolysaccharides have been removed to avoid repetition. The acronym sEPS is now used consistently throughout the manuscript without further redefinition, including revisions made in Page 6 (Paragraph 5) and Page 8, thereby improving clarity and readability.

**Response to Comment 3**: Correction of Gram Nomenclature

 The capitalization has been corrected in accordance with microbiological nomenclature. The terms Gram-positive and Gram-negative are now consistently capitalized throughout the manuscript.

**Response to Comment 4:** (Page 10 – Anticancer potential)

 Anticancer activity was predominantly associated with low-molecular-weight sEPS fractions (approximately 300–550 kDa) from *Porphyridium marinum*, generated via high-pressure homogenization (HPH), which exhibited enhanced antiproliferative effects against murine breast cancer cells, with up to 55% reduction in cell viability. ^5^

**Response to Comment 5**: 

 We thank the Reviewer for this comment. The Rheological and thermal stability section has been revised to clarify that, while high sulfation contributes to viscoelastic stability and industrial functionality, reduced molecular weight and lower viscosity can enhance biological activity by improving the accessibility of sulfate and uronic acid groups, emphasizing the need to balance rheological properties according to the intended application.
